# Noise pollution: more attention is needed

**DOI:** 10.1016/j.lanepe.2022.100577

**Published:** 2023-01-04

**Authors:** The Lancet Regional Health – Europe

Noise pollution—defined as unwanted or disturbing sounds—receives far less attention than air or water pollution because it cannot be seen, tasted, or smelled. Noise pollution is dangerous; it has negative long-term impacts on humans, marine life, and terrestrial life and hampers biodiversity. According to WHO estimates, at least 1 million healthy life-years (disability-adjusted life-years, DALYs) are lost annually from traffic-related environmental noise alone in western Europe. In 1910, the Nobel Prize winner Robert Koch predicted that “One day man will have to fight noise as fiercely as cholera and pest.” It is time that the social and environmental implications of noise pollution and its effect on public health receives more attention and becomes a priority before it becomes a planetary epidemic.

Noise is a health problem for at least one in five EU citizens. Through direct injury to the auditory system, noise can lead to hearing loss and tinnitus. Noise-induced hearing loss can be caused by a single exposure to an intense impulse sound (such as gunfire), or by steady state long-term exposure to sound pressure levels higher than L_A_ 75–85 dB—eg, in industrial settings. The trends in prevalence of tinnitus in Europe are particularly worrying—a 2021 Article published in *The Lancet Regional Health – Europe* by Roshni Biswas and colleagues estimated that more than one in seven adults in the EU have tinnitus. Beyond effects on the auditory system, noise is associated with an increased incidence of cardiovascular diseases in addition to causing annoyance, disturbed sleep, and impaired cognitive performance. It is estimated that the burden of disease, estimated as DALYs lost from environmental noise in western European countries alone, is equivalent to 61 ,000 years for ischaemic heart disease, 45, 000 years for cognitive impairment in children, 903, 000 years for sleep disturbance, 22 ,000 years for tinnitus, and 654,000 years for annoyance.

In addition to the impact of noise on humans, anthropogenic noise (eg, noise from low-flying airplanes, watercrafts, oil and gas explorations, active sonar, and seismic surveys) is a pervasive pollutant that is a threat to marine and terrestrial wildlife. Anthropogenic noise can reduce their reproductive success and increase mortality and emigration, resulting in lower population densities. Importantly, sound is essential to support marine life, which they rely on to source prey, communicate, and navigate. Increase in anthropogenic noise has a negative impact on marine creatures by decoupling their sensory perception from cognitive processes, leading to poor decisions that can often lead to death. More ambitious national and international policies are needed to regulate and deploy noise-reduction technologies to mitigate anthropogenic noise to maintain a healthy ocean.

In contrast to many other pollutants, noise can be stopped instantly and does not have lingering effects unlike other pollutants. The WHO Regional Office for Europe has developed comprehensive environmental noise guidelines for different sources of noise and for different settings that can provide guidance for local, national and international authorities. It has been shown that measures leading to decreased noise pollution in schools resulted in lower levels of annoyance and an improvement in the cognitive abilities of children. A better implementation of the Environmental Noise Directive—the main EU law to identify and address noise pollution levels—is needed to protect people from harmful exposure to environmental noise. However, one major issue is paucity of data from all countries, more specifically from central and eastern parts of the WHO European Region, which means that assessment of the burden of disease from environmental noise is not possible for the whole Region.

In addition to societal-level reforms and improved urban planning, much can be done at an individual level to minimise noise pollution. Lowering individual noise footprint is a great way to start. In the case of living in areas with roadside traffic noise, bedrooms can be moved to the quieter side of the house—noise prevents the night-time blood pressure from decreasing which is important for the body to relax. Lifestyle changes such as making low noise a lifestyle priority (when buying vehicles, air conditioners, blenders etc), seeking out quiet places, particularly on weekends and during holidays can be effective in the long term. Individuals can speak up in their workplaces, spread awareness, and educate children about the negative effects of noise pollution on health and biodiversity.

Koch's 1910 prediction acts as a warning for today: action is needed now before noise pollution reaches epidemic proportions. Similar to the CO_2_ emissions targets set at the 27th Conference of the Parties to the United Nations Framework Convention on Climate Change, national noise footprint targets are needed that countries can be held accountable to. There is a universal need for better noise policy implementation by governments.

